# A Clinicoepidemiological Study of Cutaneous and Systemic Comorbidities of Seborrheic Dermatitis in Adolescent and Adult Females

**DOI:** 10.7759/cureus.40972

**Published:** 2023-06-26

**Authors:** Rajashekar T S, Savana Waikhom, Suresh Kumar K, Meghana E Reddy

**Affiliations:** 1 Dermatology, Venereology and Leprosy, Sri Devaraj Urs Academy of Higher Education and Research, Kolar, IND; 2 Dermatology, Sri Devaraj Urs Academy of Higher Education and Research, Kolar, IND

**Keywords:** adolescents, adults, hypertension and acne vulgaris, diabetes mellitus, associated diseases

## Abstract

Background

Seborrheic dermatitis is the most common, chronic inflammatory skin condition which is confined to the scalp, nasolabial folds, and regions rich in sebaceous glands for which no definitive cause has been found. Although the disease is more common, the comorbidities associated with it have not been studied in detail. This study aims to assess the prevalence of seborrheic dermatitis and its associated cutaneous and systemic comorbidities in adolescent and adult patients.

Methodology

This cross-sectional study was performed among 451 adolescent and adult female patients who visited the Department of Dermatology, Venereology, and Leprosy of R. Laxminarayanappa Jalappa Hospital and Research Centre, Kolar. Patients having symptoms such as scaly patches, inflamed skin, and stubborn dandruff were diagnosed with seborrheic dermatitis and included in the study. A detailed history was collected for assessing other cutaneous disorders.

Results

Out of the 451 female participants, 87% belonged to the age group of 21-30 years, with 60.9% having cutaneous and 28.3% having systemic comorbidities. Acne (13.3%) and diabetes mellitus (13.1%) were the most common cutaneous and systemic associated comorbidities, respectively.

Conclusions

Comorbidities of seborrheic dermatitis were more commonly seen in adult female patients, Some of the common cutaneous comorbidities were acne, alopecia areata, and folliculitis. Systemic comorbidities included diabetes, obesity, and hypertension. However, all of these comorbidities were not statistically significant.

## Introduction

Seborrheic dermatitis, a common inflammatory disease of the skin, has a papulosquamous etiology in the region abundant in sebaceous glands such as the face, capitulum, and various bodily liquids [1]. Its prevalence ranges from 1% to 5% in the adult population, with a prevalence of as high as 23% in the female population. Globally, the prevalence of seborrheic dermatitis is around 5% among all ethnic groups [2].

Skin lesions are localized in sebum-rich areas such as the scalp, face, chest, back, and, rarely, skin folds. Although the development of seborrheic dermatitis is associated with multiple factors, its onset appears to be associated with the interaction of normal microscopic skin flora (especially, *Malassezia *spp.), the structure of lipids on the skin surface, and individual factors [3,4].

Treatment for seborrheic dermatitis remains challenging. In certain instances when it is incurable, it is controlled by topical medications and management of lifestyle [5]. Therefore, there is a need to investigate the comorbidities associated with seborrheic dermatitis which may contribute to clarifying the pathological mechanism of the disease and the advancement of new therapeutic approaches.

Only women were included in this study as they are more cautious about their facial appearance and hair patterns than men [6].

Even though it is a common disorder, only limited studies have been documented on the generality of seborrhoeic dermatitis and its incidence in correlation with other skin and systemic diseases [7]. Because there is a paucity of the literature on the association of comorbid conditions with seborrheic dermatitis, the present study aimed to assess the cutaneous and systemic comorbidities in adolescent and adulthood females. Adolescence is defined as the period between the onset of puberty and adulthood is considered as the period between adolescence and old age.

## Materials and methods

This cross-sectional, observational study was conducted in the Department of Dermatology, Venereology, and Leprosy of R. Laxminarayanappa Jalappa Hospital and Research Centre, Kolar between February 2022 and November 2022. The study was approved by the Institutional Ethical Committee (approval number: DMC/KLR/IEC/723/2020-21). All female patients aged between 14 and 70 years who were diagnosed with seborrheic dermatitis with symptoms of scaly patches, inflamed skin, and stubborn dandruff and were willing to participate were considered in this study. All pregnant women were excluded from the study. A detailed history and complete clinical examination were obtained, and the results were noted.

## Results

**Figure 1:**
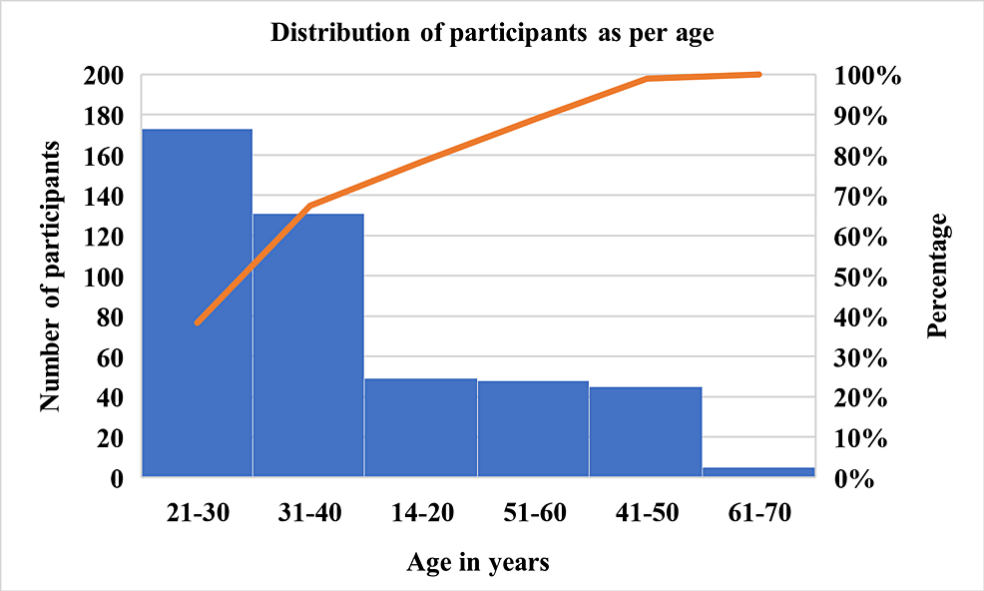
Number of participants distributed according to age.

The correlation between cutaneous comorbidities and seborrheic dermatitis was assessed using the Pearson correlation test and was found to have a significant negative correlation. A total of 451 female participants were included in this study. The age of the participants ranged from 14 to 70 years with a prevalence of 87% in the age group of 21-30 years, 65% in the age group of 31-40 years, 23% in the age groups of 14-20 and 51-60 years, 21% in the age group of 41-50 years, and 3% in the age group of 61-70 years, as shown in Figure 1.

**Figure 2:**
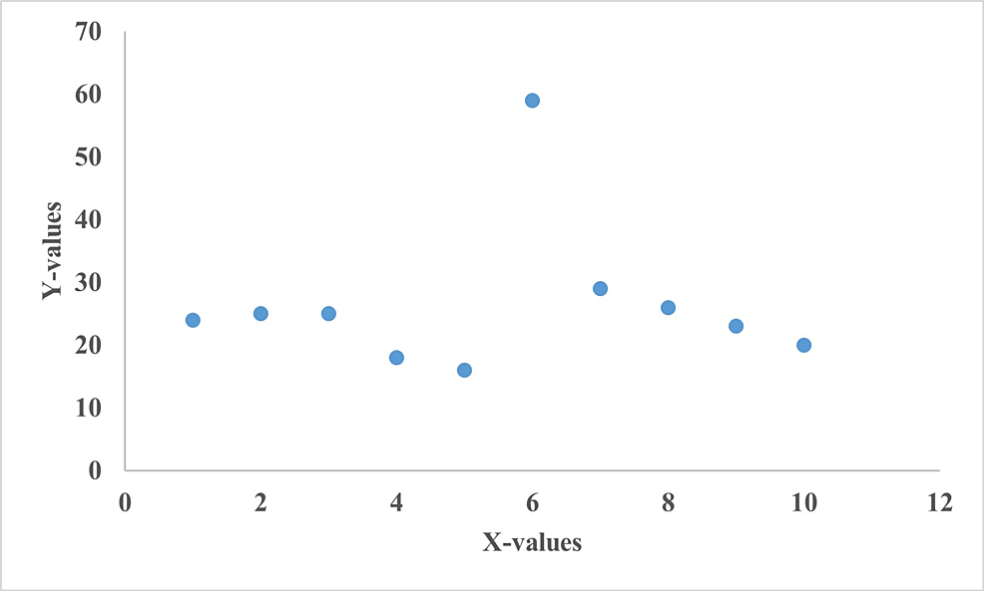
Correlation between comorbidities associated with seborrheic dermatitis.

The Pearson correlation test was performed between systemic and cutaneous comorbidities which showed a significant negative correlation (Figure 2).

**Figure 3:**
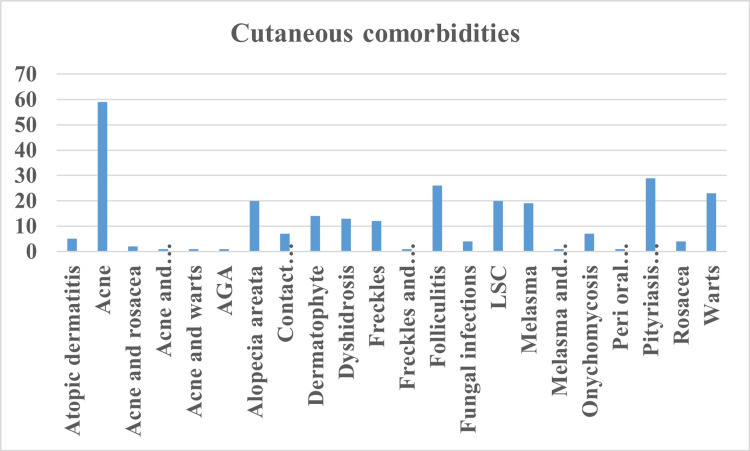
Cutaneous comorbidities associated with seborrheic dermatitis. AGA: androgenetic alopecia; LSC: lichen simplex chronicus

In total, 59 participants had acne, followed by warts in 32 patients, pityriasis versicolor in 29 patients, folliculitis in 26 patients, warts in 23 patients, lichen simplex chronicus in 20 patients, and alopecia areata in 20 patients. The detailed cutaneous comorbidities associated with seborrheic dermatitis are illustrated in Figure 3.

In total, diabetes and hypertension followed by diabetes and obesity accounted for 0.05% each, and 72 (15.9%) patients had overlapping cutaneous and systemic comorbidities. The detailed comorbidities are presented in Table 1.

**Table 1:** Systemic comorbidities associated with seborrheic dermatitis.

Systemic assessment	Number of participants	Percentage of total population (n = 451)
Diabetes mellitus	14	3.1%
Diabetes and obesity	25	5.2%
Diabetes, hypertension, and Obesity	1	0.2%
Hypertension and diabetes	25	5.2%
Hypertension	24	5.1%
Hypertension and obesity	1	0.2%
Obesity	18	3.5%
Thyroid diseases	16	3.2%

## Discussion

Seborrheic dermatitis is a common, chronic relapsing skin disease affecting the seborrheic areas of the body which is characterized by the appearance of red, flaking, greasy areas of the skin. In normal patients, seborrheic dermatitis may be coincidental with dermatological conditions such as acne, rosacea, pityriasis versicolor, pityrosporum folliculitis, and other systemic diseases such as HIV, Parkinson’s disease, mood disorders, alcoholic pancreatitis, and Down’s syndrome [8].

The study aimed to investigate the comorbidities associated with seborrheic dermatitis in adolescent and adult females. When compared with adolescents, seborrheic dermatitis was more prevalent in adult participants, which is not represented in the literature but is evident in our study observation.

Few studies have reported that acne, folliculitis, and rosacea are cutaneous comorbidities associated with seborrheic dermatitis, which is consistent with the present study where acne folliculitis and rosacea were a few of the comorbidities with a low incidence (Table 1) [9-12]. These findings indicate that the cutaneous microbiological agreement, lipid regularity, and seborrhoea can likely play a crucial role in the pathogenesis of the disease.

Furthermore, these patients show a significantly higher percentage of human papillomavirus infection (7.1%) and alopecia areata contrastingly compared to previous studies [12,13]. This fact prompts speculations regarding whether common immunological dysfunction predisposes patients to both skin conditions.

In this study, the common systemic comorbidities associated with seborrheic dermatitis were hypertension and diabetes (13.9% and 11.3%) similar to a previous cross-sectional study [13]. This may be because *Malassezia* yeast enhances the issue of inflammatory cytokines such as interleukin (IL)-6, IL-8, and tumor necrosis factor α (TNF-α) keratinocytes which cause concomitant upregulation of the Ang-II type I receptor and transforming growth factor-β1 which leads to the pathogenesis of hypertension [14]. One of the components of the pathogenesis of obesity is inflammation, and many inflammatory cytokines such as C-reactive protein, IL-6, and TNF-α play a vital role in the disease and are also commonly raised in seborrheic dermatitis patients [15].

## Conclusions

This study emphasizes the importance of screening associated cutaneous and systemic comorbidities in patients with seborrheic dermatitis to treat them simultaneously with seborrheic dermatitis to improve their quality of life. The more prevalent conditions noted in cutaneous comorbidities include acne, folliculitis, and alopecia areata, and prevalent systemic comorbidities include hypertension, obesity, and diabetes.
